# Failure induces task-irrelevant exploration during a stencil task

**DOI:** 10.1007/s00221-023-06548-2

**Published:** 2023-01-20

**Authors:** Katinka van der Kooij, Nina M. van Mastrigt, Joshua G. A. Cashaback

**Affiliations:** 1 Department of Human Movement Sciences, Vrije Universiteit Amsterdam, Amsterdam, Netherlands; 2 Biomechanics and Movement Science Program, Kinesiology and Applied Physiology, Interdisciplinary Neuroscience Program, University of Delaware, Biomedical Engineering, Mechanical Engineering, Newark, DE USA

**Keywords:** Motor learning, Reward, Exploration, Variability

## Abstract

During reward-based motor tasks, performance failure leads to an increase in movement variability along task-relevant dimensions. These increases in movement variability are indicative of exploratory behaviour in search of a better, more successful motor action. It is unclear whether failure also induces exploration along task-irrelevant dimensions that do not influence performance. In this study, we ask whether participants would explore the task-irrelevant dimension while they performed a stencil task. With a stylus, participants applied downward, normal force that influenced whether they received reward (task-relevant) as they simultaneously made erasing-like movement patterns along the tablet that did not influence performance (task-irrelevant). In this task, the movement pattern was analyzed as the distribution of movement directions within a movement. The results showed significant exploration of task-relevant force and task-irrelevant movement patterns. We conclude that failure can induce additional movement variability along a task-irrelevant dimension.

## Introduction

Reward-based motor adaptation depends on learning from success (reward) and failure (reward absence) (Cashaback et al. 2017; Izawa and Shadmehr 2011; Therrien et al. 2016; Van der Kooij and Smeets 2019). The general principle is that on the next attempt rewarded motor commands are maintained whereas non-rewarded motor commands are varied. In other words, reward induces exploitation whereas reward absence induces exploration. Exploration involves changing the motor commands (Cashaback et al. 2017; Izawa and Shadmehr 2011; Therrien et al. 2016). Therefore, exploration causes variability in the movements in addition to inevitable variability that arises due to noise in the perception of target, in the planning of the movements, or in the execution of the movement (‘sensorimotor noise’). The key difference between exploration and other sources of variability is that the exploration is aimed at improving performance (Dhawale et al. 2017; Mastrigt et al. 2021; Therrien et al. 2018), unlike sensorimotor noise. As failure induces the need to improve performance, exploration can be studied as failure-induced variability (Dhawale et al. 2017; Mastrigt et al. 2021; Therrien et al. 2018). For simplicity we refer to all other sources of variability other than failure-induced variability as sensorimotor noise (Mastrigt et al. 2021). This rationale is consistent with current models of reward-based learning that add exploration as a source of movement variability to the movement plan, which is modulated according to reward-history (Cashaback et al. 2019; Dhawale et al. 2017; Therrien et al. 2018).

When only considering the aspect of the movement that influences success, for instance the reach direction in a reaching task, the mechanism of exploration–exploitation seems straightforward. Failure enhances movement variability along a dimension that is explicitly rewarded, and this movement variability is used to adapt the movement plan along the relevant dimension. Indeed, failure induces (i) changes in reach direction when reaching in a direction from a central starting point (Cashaback et al. 2019; Pekny et al. 2015; Sidarta et al. 2016; Therrien et al. 2016; Uehara et al. 2019), (ii) changes in the reach trajectory when matching a trajectory (Brummelman et al. 2022; Chen et al. 2017; van der Kooij et al. 2021b), and (iii) changes in the centre of pressure trajectory when performing a balance task (van Mastrigt et al. 2020).

The mechanism of exploitation and exploration is less straightforward when considering that in most tasks not all dimensions of the movement influence success. Generally, some dimensions of the movement are task-relevant, whereas others are task-irrelevant (redundant dimensions, or ‘uncontrolled manifolds’ (Latash et al. 2002)). Such task-relevance can be defined in task space (Nashed et al. 2012), joint space (Latash et al. 2002), or muscle space (Valero-Cuevas et al. 2009). As the task-irrelevant dimensions do not affect performance, it has been proposed that the nervous system does not control variability along these dimensions by making online corrections to sensory feedback about the movements (Nashed et al. 2012; Valero-Cuevas et al. 2009) or by adjusting the movement plans (Abram et al. 2022; Rebelo Dal’Bello and Izawa 2021; van Beers et al. 2013). This ‘minimal intervention principle’ (Todorov and Jordan 2002) has been observed in many tasks, ranging from reaching (Nashed et al. 2012; van Beers et al. 2013), walking (Abram et al. 2022), hand gestures (Rebelo Dal’Bello and Izawa 2021), to force production (Valero-Cuevas et al. 2009). It is unclear however whether task-irrelevant dimensions are explored. If there is no influence on task success along task-irrelevant dimensions, there might be no reason to explore. On the other hand, one might argue that if there is no reason to mitigate movement variability, there is no reason to *not* explore.

The main question that we focus on is whether task-irrelevant dimensions are explored during a reward-based motor learning task. Although the task-irrelevant dimension does not affect task performance, there might be both advantages and disadvantages to exploring task-irrelevant dimensions.

Exploring task-irrelevant dimensions might be advantageous because it allows the learner to discover the structure of a task (Rebelo Dal’Bello and Izawa 2021) which might be initially unknown or change over time. For instance, when grasping a bottle, the reach direction is clearly task-relevant while it might be initially unknown whether the reach height affects the ability to drink from the bottle. In addition, other costs might be discovered such as the energy cost involved in lifting the bottle. Moreover, while the fluid in the bottle reduces because you drink from it, the relation between reach height, reach success and energy costs might change. Although sensorimotor noise might accidentally lead one to a better movement, exploration might be beneficial because exploration has the feature that it can be learned from, in contrast to other sources of variability, such as inevitable sensorimotor noise (Therrien et al. 2016, 2018).

On the other hand, exploration in task-irrelevant dimensions might be disadvantageous because it increases the complexity of the learning problem. If one is successful following a change in reach direction, it is evident that the reach direction should be maintained if no other changes have been made. For example, when both reach direction and movement extent are changed, it is unclear whether both should be maintained or only one of the two. Reward-based motor learning, a type of reinforcement learning, suffers from a curse of dimensionality (Sutton and Barto 2018 in (Niv et al. 2015). Consistently, reward-based motor learning has been found more difficult in a three-dimensional task compared to a one-dimensional task (van der Kooij and Smeets 2019). To solve this complexity problem, the nervous system might determine task-relevance to focus only on task-relevant dimensions (Niv et al. 2015).

To date, variability during a reward-based motor learning task has been shown to depend on task-relevance. In contrast to the observations of larger variability in task-irrelevant dimensions, variability has been found to increase with task-relevance during reward-based motor learning. In a forcefield adaptation task, position variability increased when the forcefield was position-dependent and velocity variability increased when the forcefield was velocity-dependent (Wu and Miyamoto 2014). As the variability increases in response to an increase in task-relevance, the variability might reflect exploration rather than sensorimotor noise. Although these results on variability support the idea that exploration depends on task-relevance, the studies did not focus on failure-induced exploration. Therefore, it is unclear whether task-irrelevant dimensions are explored to some extent or whether these dimensions remain unexplored.

To answer the question whether task-irrelevant dimensions are explored, variability due to exploration needs to be separated from sensorimotor noise (He et al. 2016). To separate the exploration from sensorimotor noise, exploration can be measured as failure-induced movement variability (Mastrigt et al. 2021; Pekny et al. 2015). In this study, we ask whether task-irrelevant dimensions are explored, and measure exploration as failure-induced variability. To this end, we use a task in which participants are asked to colour visual targets green by making an erasing movement with the correct force with a force-sensitive stylus on a drawing tablet. In this task, the force is task-relevant whereas the spatial pattern of the erasing movement is task-irrelevant.

## Methods

### Participants

We based the power analysis on a within-group comparison of variability following success and variability following failure. Previous studies showed a large effect size for the effect of failure on the variability in the task-relevant dimension (van der Kooij and Smeets 2019; van der Kooij et al. 2018; van Mastrigt et al. 2021). The effect of failure on variability in task-irrelevant dimensions is to the best of our knowledge unknown. We aimed for detecting a moderate effect size. Power analysis with G*Power showed that for a moderate effect size (*d* = 0.5), and 80% statistical power (Beta = 0.2), alpha = 0.05, 27 participants are needed in the paired-samples *t*-test to compare variability in the non-rewarded dimension following reward and failure. Due to the COVID-19 pandemic, we measured only 22 participants (16 females, 4 left-handed; 22 ± 3 years old). Participants studied at the faculty of Behavioural and Movement Sciences of the Vrije Universiteit Amsterdam and received either course credit or 30€ for their participation.

### Materials

**Fig. 1:**
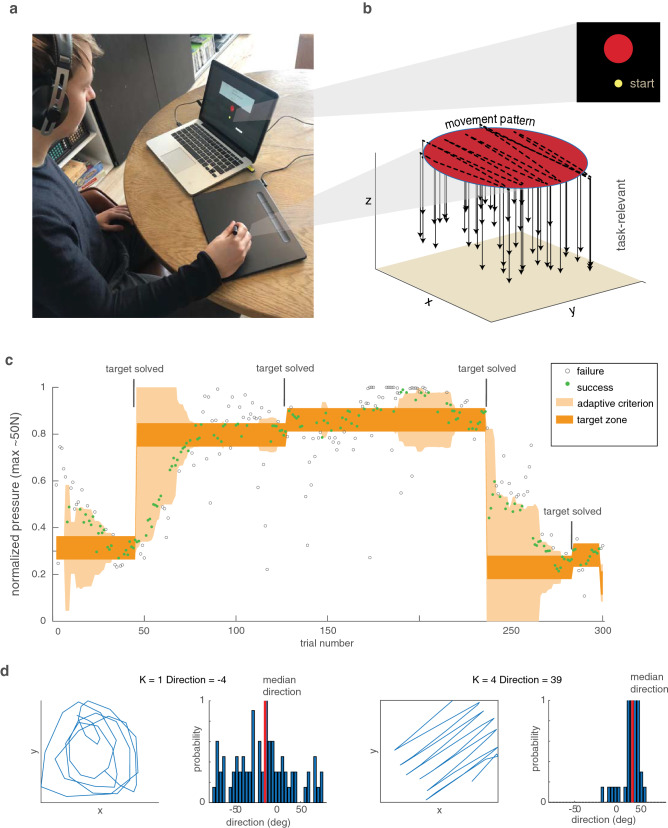
Methods. **a** Experimental set-up with Wacom device and computer. **b** Example data from a single trial with the task-relevant dimension (force, arrows) and the task-irrelevant dimension (movement pattern, dotted line). **c** Example 300-trial session of a participant that illustrates different targets, reward criterion, and task-relevant force. Trials defined as a failure are open symbols and trials defined as a success are green. **d** Two different movement patterns with corresponding distributions of movement directions. The back-and-forth (‘striped’) pattern shows a higher concentration of movement directions than the circular pattern

Participants performed the task at home on their laptop or PC, along with a Wacom Intuos Medium tablet that we used for detection of the force and movement pattern of the erasing movement. The stencil task was developed with the Unity3D game engine and can be downloaded from https://osf.io/tcba9/. The Wacom device measured the axial force on the stylus with a spring in the stylus and measured the position of the stylus on the tablet with sensors in the tablet. Both the axial force and position were sampled at 90 Hz. The maximum force detected by the Wacom stylus was about 50 Newton. Participants viewed visual target stimuli on the screen and made erasing movements on the tablet (Fig. 1a). To control for differences in the pixel size of different monitors, visual stimuli and spatial data were scaled by the monitor’s points per inch.

### Task

We instructed participants to score points by erasing small circular targets (diameter: 1 cm; Fig. 1a) with the stylus, using the right amount of force. This way, they were explicitly instructed that force was the task-relevant dimension while receiving no instructions about the task-irrelevant dimension of movement pattern. The tablet on which participants moved was smooth. Hence there was no intrinsic relation between the axial force on the stylus and the acceleration on the tablet. To ensure that we could measure a movement pattern, participants were asked to cover the entire surface of the target with their erasing movement. When the average movement velocity in a trial was below 1 cm per second, a beep sounded, the participant could not score points, and received a visual text reminder: ‘Please rub the entire target.’ We excluded one participant for whom this happened on more than 10% of the trials from the data analysis. Across the other participants, this occurred in less than 0.01% of the trials. We did not inform participants about the target force but participants did see the spatial position of the Wacom pen represented with a standard cursor on the computer screen.

The target was presented against a grey background plane. So that different targets could be identified, the colour of the target changed with the target force. When the target colour changed, the target colour was selected pseudo-randomly from RGB colour space such that it differed at least 0.2 points in normalized space from the background colour and feedback colours (red or green).

To start a trial, the participant moved the cursor representing the stylus position into a small yellow start position 1 cm below the target circle (Fig. 1b). After that, a new target circle appeared. Once the cursor touched the target circle, the target stayed on the screen for two more seconds so that participants could perform an erasing movement. After the two seconds, we provided feedback for 500 ms based on the mean force during the last 1,5 s of the erasing movement. We refer to this mean force during this period as the applied force.

The participant’s task was to minimize the force error: the absolute difference between the target zone and the applied force. To help participants reduce the force errors, we used an adaptive (‘closed loop’) reward criterion (Therrien et al. 2016). The adaptive criterion (Fig. 1c) rewarded force errors smaller than a moving 30th percentile of the past 10 trials. When a trial was rewarded, the target turned green. Otherwise, it turned red. After a 500 ms inter-trial interval, the next trial started.

When eight out of the past ten trials had hit the target zone, a new target appeared that required a different, randomly chosen, target force. To inform the participant about the target change, the target changed colour. Before each target change, the participant was asked to rate motivation with a Quick Motivation Index (Van der Kooij et al. 2021a) that asked the participant to respond to the following two questions:

- How much did you enjoy the task until now?
- How motivated are you to continue?

For this, they used two sliders ranging from ‘not at all’ to ‘very much’, which was initiated at the lower ‘not at all’ end. We collected these ratings for a separate research line on motivation as a function of success frequency (van der Kooij et al. 2021a).

### Procedure

The experiment started with a video call in which the experimenter supervised the installation of the experimental code and Wacom driver. The experimenter also showed an instruction video, which placed the experiment in the context of a conservator cleaning an art object. When the participant understood the task, the video call was stopped, and the participant independently completed the five experimental sessions, which on average each lasted 23 min. The experimenter instructed participants to leave one night of sleep between experimental sessions and informed them that they could skip one day and catch up the next day.

### Data analysis

The datasets generated and analyzed during the current study are available in the Open Science Foundation repository: https://osf.io/tcba9/. Kinetic and kinematic data, consisting of force and spatial coordinates per trial, were filtered using a bi-directional, second-order, low-pass Butterworth filter with a cut-off frequency of 20 Hertz. We defined two primary dependent variables: the force exploration and the pattern exploration.

Force exploration was derived from the axial force on the stylus. This was registered in Wacom units, normalized by the standard Wacom software to the full force detection range which was about 0–50 Newton. For each trial, we measured the mean force during the last 1,5 s of the erasing movement.

Pattern exploration was derived from the positional data of the stylus. The position of the stylus was registered in screen coordinates which were transformed into centimetres using the device’s points per inch (ppi). Participants could alternate between more striped and more circular patterns. These pattern differences can be captured by the distribution of movement directions. Based on the position of the stylus during the erasing movement we expressed the pattern in each trial by a distribution of movement directions. We calculated the movement direction in each of the ~ 135 samples during the 1,5 s movement by taking the derivative of lateral (*x*) and vertical (*y*) positions on the tablet and taking the arc tangent of d*x*/d*t* divided by d*y*/d*t*. This way, back-and-forth movements could be defined by a single direction because it provides the same sign for movement directions in opposite quadrants. For left-handed participants, movement directions were flipped along the y-axis.

The force and pattern exploration were both derived from a variability measure based on a trial-by-trial method suitable for studying variability during learning (Pekny et al. 2015). Since we study exploration as failure-induced variability, the analysis was built on subtracting trial-by-trial changes following failure and following success. In this analysis, we assume that variability following success reflects sensorimotor noise whereas variability following failure reflects both sensorimotor noise and exploration. Moreover, we assume that sensorimotor noise and exploration are independent sources of variability. A pitfall in retrieving exploration by subtracting sensorimotor noise is that the performance-dependent feedback causes correlated samples of motor noise and exploration (Mastrigt et al. 2021). This sampling bias might be overcome by using the previous trial. When trial t is successful, the previous trial *t* – 1 isn’t necessarily a successful trial. Simulation results showed that, when using a success criterion similar to the one we used, the pitfall can indeed be circumvented by comparing the trial following the successful or unsuccessful trial (*t* + 1) with the trial before the successful or unsuccessful trial (*t* – 1) rather than comparing trial *t* + 1 and the successful or unsuccessful trial itself (*t*) (Mastrigt et al. 2021). Although this problem does not occur for the task-irrelevant dimension, we used the same method for symmetry between the two analyses.

For each dependent variable $z$ (either force or pattern), we calculated the absolute changes $\delta$ as follows:

$$\delta = \left|{z}_{t+1}-{z}_{t-1}\right|$$

Using these equations, we then built up the set of changes following failure (${\Delta }_{f}$) and following success (${\Delta }_{s}$). We calculated the set both for the entire experiment and for each session separately. For both force and pattern, exploration was defined as the fraction increase in changes following failure relative to changes following success. Exploration $\eta$ in the dependent variable was calculated as follows, with $M$() denoting the median:

$$\eta = \frac{M{(\Delta }_{f}){-M(\Delta }_{s})}{{M(\Delta }_{s})}$$

A value of $\eta$ greater than 0 corresponds with greater exploratory behaviour.

Force changes were expressed as a difference in force between two erasing movements. Pattern changes were expressed as a difference in distributions of movement directions in the movement patterns of two erasing movements (i.e. trials). We used a Wasserstein distance (Vallender 1974) to quantify the difference between the distributions of movement directions. This Wasserstein distance captures the transformation of one distribution into the other. We defined distance as the angular distance on the ground torus. Larger values indicate larger differences in movement pattern.

We calculated exploration in force and pattern both across sessions to test our primary hypothesis that failure induces exploration, and for each session separately to test whether exploration changed with the session.

Task performance was defined as the number of targets solved per session.

### Preparatory analyses

To check whether parametric *t*-tests suit the data, we visually assessed the distribution of median force changes and median pattern changes over individuals using histograms and QQ-plots. When the data deviated from a normal distribution, planned *t*-tests for pattern changes and force changes were replaced with their non-parametric equivalent: Mann–Whitney-*U* tests for between-group comparisons and Wilcoxon rank sum tests for within-participant comparisons.

**Fig. 2:**
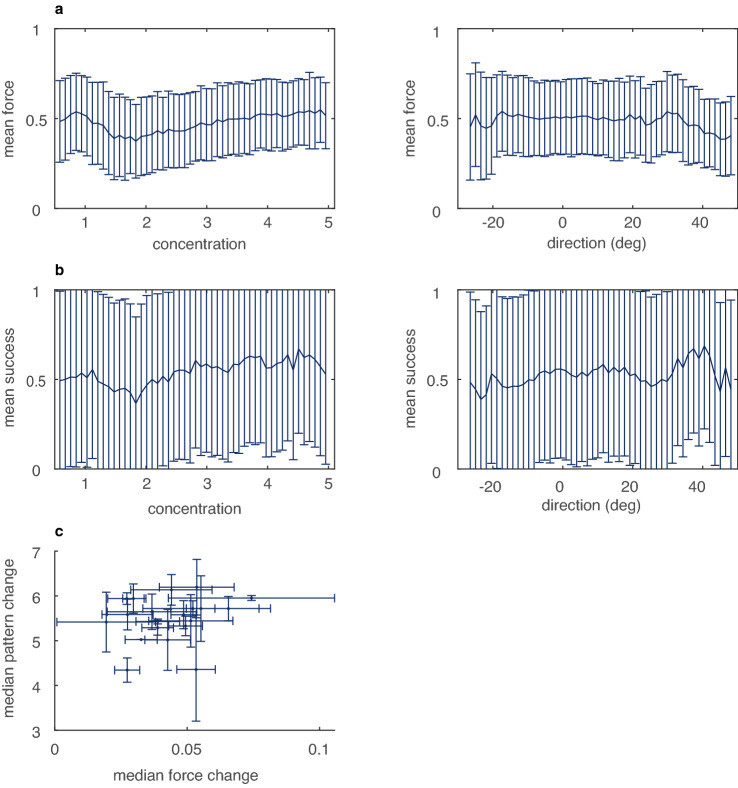
Task relevance of the movement pattern. All graphs display statistics calculated over all trials of all five sessions. **a** Movement pattern is not related to force. Mean force with standard deviation for 50 equally sized bins of concentration of the movement pattern (left panel) and direction of the movement pattern (right panel). **b** Movement pattern is not related to success. Mean success with standard deviation for 50 equally sized bins of concentration of the movement pattern (left panel) and direction of the movement pattern (right panel). **c** Median pattern change with interquartile range as a function of median force change with interquartile range

To check our assumption that force was the only task-relevant parameter, we analyzed the task relevance of the movement pattern. To do so, we summarized each movement pattern with a Von Mises distribution of movement directions. This distribution has two parameters: the concentration (Fig. 2a) and the median direction (Fig. 2b). A high concentration is related to a striped pattern whereas a low concentration is related to a more circular or random pattern (Fig. 2a). To assess the task-relevance of these parameters, we correlated them with the applied force, the task-relevant parameter. We did this across trials to test whether certain patterns were more suited for certain forces, and within trials to test whether certain patterns were related to variations in force within a movement. In addition, we correlated the two movement pattern parameters with tangential acceleration along the plane of the tablet. We did so because acceleration on the tablet requires force which might affect the force on the stylus.

For the across-trial analyses, we calculated one Spearman correlation per parameter per participant. For the within-trial analyses, we calculated one Spearman correlation per trial and averaged them over all trials per participant.

As a last check to our assumption that the movement pattern is task-irrelevant, we tested the independence of force variability and pattern variability, by correlating the average task-relevant force changes and the task-irrelevant changes in movement pattern (Rebelo Dal’Bello and Izawa 2021).

### Statistical tests

We first tested the assumption that failure induces exploration in the task-relevant dimension. To this end, we performed a one-sample signed rank test on the force exploration. Next, we tested the hypothesis that failure induces task-irrelevant exploration by performing a one-sample signed rank test on the pattern exploration. As an exploratory analysis, we assessed how force and pattern exploration changed over time. We expected that if task-irrelevant exploration does not benefit performance, pattern exploration would decrease over time. To test this, we entered the force and pattern exploration in a multilevel regression with the session as a fixed effect and with a random intercept per participant:

$${\text{Exploration}}\sim {\text{session}} + \left( {1\left| {{\text{participant}}} \right.} \right)$$

Significance of fixed effects was tested using *t*-tests.

## Results

As participants performed the task independently at home, some participants returned the equipment with missing sessions. In total 3.6% of sessions were missed and those were excluded from data analysis.

Participants were able to learn the task. On average, participants needed about 78 trials to resolve a target and thus on average solved 3.8 ± 1.5 targets per session.

The preparatory analyses support the idea that the movement pattern was task-irrelevant. Neither concentration of the movement pattern (*p* = – 0.02), nor median direction of the movement pattern (*p*  = – 0.08) was related to the mean applied force (Fig. 2a). Neither were the concentration of the movement pattern *p* = – 0.06) or the direction of the movement pattern (*p* =  – 0.05) related to the mean success (Fig. 2b). As expected, the concentration was related to average acceleration, indicating that movement patterns with a high concentration (e.g. striped patterns) had a higher acceleration than movements with a low concentration (e.g. circular patterns). However, acceleration and the axial force that the participant exerted with the stylus were not related. Within a movement pattern, the median ρ between acceleration and force across participants and trials was – 0.01. Furthermore, variability in force and variability in movement pattern were not related (*p* = – 0.17; Fig. 2c).

**Fig. 3:**
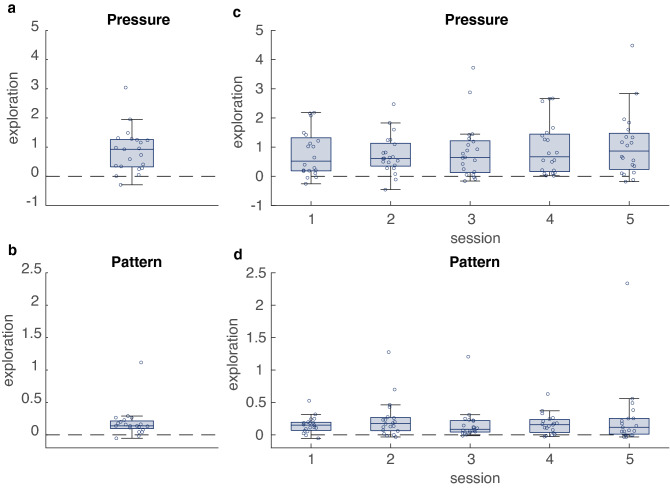
Exploration of force and movement pattern, measured as the fraction increase in variability following failure. Values greater than 0 (dashed line) correspond to exploration. **a** Force exploration. **b** Pattern exploration. **c** Force exploration as a function of the session. **d** Pattern exploration as a function of the session. Dots represent individual data points

Participants showed exploration in both task-relevant and task-irrelevant parameters. One-sample signed rank tests showed significant force exploration (*z* = 3.84, *p* < 0.01; Fig. 3a) and also significant pattern exploration (*z* = 3.73, *p* < 0.01; Fig. 3b). We then tested whether force exploration and pattern exploration changed over time. Force exploration increased with session (*B* = 0.09 CI [0.03, 0.16], *p* < 0.01; Fig. 3c). Pattern exploration, in contrast, did not depend on session (*B* = 0.02 CI [ – 0.01, 0.04], *p* = 0.3; Fig. 3d). These results were robust to outlier removal, where we excluded data points that deviated more than three standard deviations away from the mean (force exploration: *B* = 0.07 CI [0.01, 0.13], *p* = 0.02; pattern exploration: *B* = 0.02 CI [ – 0.01, 0.04], *p* = 0.30).

### Additional analyses

In a set of additional analyses, we studied learning of target forces and movement pattern, and dependency of variability on a longer success history than the previous trial.

The median number of targets solved did not change from session 1 to session 5 (Medians: 3, IQR 2.25 and 4 IQR 4, *p* = 0.22), indicating that participants did not improve their performance. Neither did the median direction of the movement pattern change from session 1 to session 5 (Medians  – 0.19, IQR 7.57 and 5.27, IQR 14.33, *p* = 0.33). The median concentration of the movement pattern reduced slightly from session 1 to session 5 (Medians 0.99, IQR 1.78 and 1.31 IQR 2.02, *p* < 0.01).

Finally, we analyzed how the pattern and pressure changes depended on a longer history of success and failure, as quantified with the success rate ${{\overline{r}} }_{t}$. We calculated the success rate as in (Dhawale et al. 2017).

$${{\overline{r}} }_{t}= {\overline{r} }_{t-1}+\left(1-\mathrm{exp}\left(\frac{-1}{\tau }\right)\right){\delta }_{t-1}$$

**Fig. 4:**
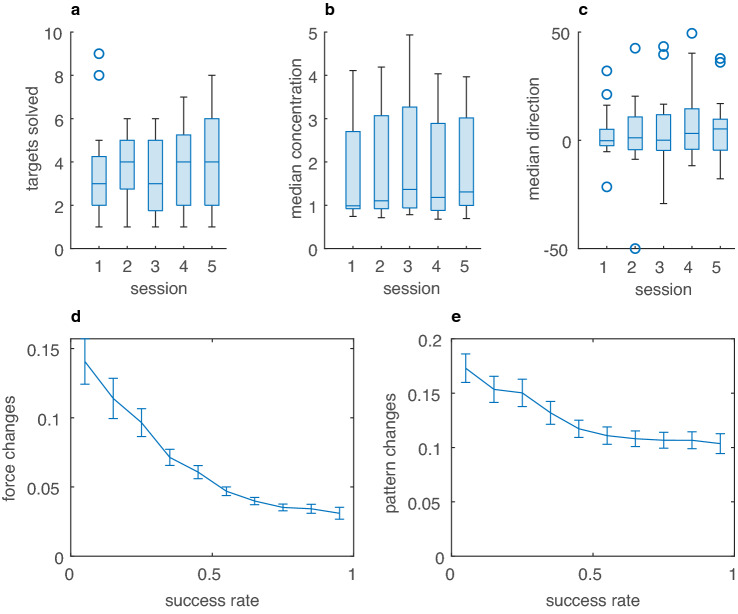
Learning and dependency of trial-by-trial changes ($\updelta$) on success rate. **a** Boxplot of median number of targets solved in the different sessions. **b** Boxplot of the median concentration in the different sessions. **c** Boxplot of the median direction in the different sessions. **d** Mean median force changes of participants (across sessions and trials) as a function of success rate with standard errors of the mean. **e** Mean median pattern changes of participants (across sessions and trials) with standard error of the mean

In this equation, ${\delta }_{t}$ is the reward prediction error (${r}_{t}- {\overline{r} }_{t-1}$) and $\tau$ (4.9; Dhawale et al. 2017) is a time constant. To assess the relation between success frequency and changes in force and pattern, we calculated the mean change with standard deviation for ten equally sized success frequency bins. We used the time constant of 4.9 estimated in (Dhawale et al. 2017). The force and pattern changes depend in a similar manner on the success rate (Fig. 4).

## Discussion

We asked whether failure induces exploration of a task-irrelevant dimension in a reward-based motor learning task. To this end, we used a stencil task in which the force was task-relevant and the movement pattern of the erasing movement was task-irrelevant. We found that participants adjusted the force they used in response to the reward feedback. On average they needed about 78 trials to discover the force with which they could resolve a target. The fact that participants learned the task shows the versatility of reward-based learning beyond reaching (Chen et al. 2017; Codol et al. 2020; Dam et al. 2013; Izawa and Shadmehr 2011; Therrien et al. 2016; 2018; Van der Kooij and Smeets 2019), pinch force tasks (Vassiliadis et al. 2021), and split-belt adaptation (Roemmich and Bastian 2015). Importantly, we found not only force exploration but also pattern exploration, indicating that both task-relevant and the task-irrelevant dimension were explored. Force exploration increased slightly over the five sessions, but pattern exploration remained constant over time.

Two study limitations are important for the interpretation of the results. First, we analyzed task-relevant and task-irrelevant exploration in different units (force and direction). Therefore, we cannot directly compare exploration between these dimensions. Future studies will have to determine whether task-relevant exploration increases over time whereas task-irrelevant exploration remains stable over time. Second, we analyzed task-irrelevant exploration in a task that we framed as an erasing task which might have caused participants to perceive the movement pattern to be task-relevant. When erasing a stain from a surface in daily life, the movement pattern might be task-relevant. For instance because the axial force produces frixtion on the surface, or because it is easier to cover the entire surface with either a striped or circular pattern.

In our experimental task, the movement pattern was not systematically related to performance in terms of force error or in terms of success. Moreover, pattern variability and force variability were not related, suggesting that they were independent sources of variability. This is in line with other results that task-relevant and task-irrelevant variability are not related (Rebelo Dal’Bello and Izawa 2021). The movement pattern was related to acceleration, but within an erasing movement, the acceleration was not related to the axial force that the participant exerted. Although there was no systematic relation between pattern and force across trials and sessions, pattern and force might have been related in a more complex manner that depended on the participant and the trial. We did find a change towards patterns with a higher concentration over the repeated sessions, which suggests that some other benefit than task-success might have been associated with patterns with a higher concentration. Perhaps the back-and-forth movement pattern is a simpler movement than the circular movement pattern.

Together, the results demonstrate failure-induced exploration in a task-irrelevant dimension. This shows that, although exploration is tuned towards task relevance (Vassiliadis et al. 2021; Wu and Miyamoto 2014), the task-irrelevant dimensions are explored. Our findings add to the well-established finding that variability in non-rewarded dimensions is maintained (Latash et al. 2002; Muller and Sternad 2009; Todorov and Jordan 2002; Valero-Cuevas et al. 2009; van Beers et al. 2013). We show that in addition to maintenance of inevitable variability (sensorimotor noise), variability is increased following failure.

Task-irrelevant dimensions might be explored to maintain performance in the face of volatile task relevance. For instance, in our task, the acceleration was not related to the force because the stylus registered the axial force, and the tablet was flat. When a surface is textured or curved, acceleration and force on the surface will be related because the lateral acceleration produces forces onto the surface. Moreover, the task-irrelevant exploration was not detrimental to performance as the task-relevant and task-irrelevant variability were not related. Consistently, it has been suggested before that variability in task-irrelevant dimensions can be used to discover task-relevance (Todorov and Jordan 2002) and to discover better motor commands (van Beers et al. 2013). Following this rationale, task-irrelevant exploration might depend on the extent to which task-relevance is plausible.

In the Introduction, we dissociated two sources of variability: exploration aimed at learning and inevitable sensorimotor noise. Sensorimotor noise arises from noise in the perception of a movement target, noise in the execution of a movement, and noise in the planning of the movement (He et al. 2016). Although these sources of variability are not aimed at learning, the nervous system might be able to learn from other sources of variability than exploration. In particular planning noise might be learned from. One study showed that planning noise in a task-irrelevant dimension accumulates across movements, suggesting that it is represented in the system and can be learned from (van Beers et al. 2013). The nervous system might add exploration to inevitable planning noise because the amplitude of planning noise might be small relative to potential changes in task-relevance.

## Conclusion

We found failure-induced variability in a task-irrelevant dimensions. This shows that these dimensions are actively explored. Important questions for future research are whether this task-irrelevant exploration benefits performance upon a change in task relevance.

## Data Availability

The datasets generated and analyzed during the current study are available in the Open Science Foundation repository: https://osf.io/tcba9/.
